# Boron‐Catalyzed Polymerization of Dienyltriphenylarsonium Ylides: On the Way to Pure C5 Polymerization

**DOI:** 10.1002/anie.202015217

**Published:** 2021-03-01

**Authors:** Xin Wang, Nikos Hadjichristidis

**Affiliations:** ^1^ Physical Sciences and Engineering Division KAUST Catalysis Center Polymer Synthesis Laboratory King Abdullah University of Science and Technology (KAUST) Thuwal 23955 Saudi Arabia

**Keywords:** arsonium, boranes, polymers, reaction mechanisms, ylides

## Abstract

The first C5 polymerization is reported, where the main‐chain is growing by five carbon atoms of the monomer at a time. Three dienyltriphenylarsonium ylide monomers were synthesized and polymerized with triethylborane as an initiator, leading to random terpolymers (C1, C3, C5) with mainly C5 repeating units (up to 84.1 %). It has been found that the methyl group (electron‐donating substituent) on the conjugated double bond of the ylides facilitates the formation of C5 segments. A mechanism was proposed based on NMR characterization and DFT calculations. The high C5 content ensures that things are on the right track for pure C5 homopolymerization.

## Graphical abstract

Reported for the first time is an unusual borane‐initiated polymerization of arsonium ylide monomers, where the main‐chain grows by five carbon atoms, from the monomer, at a time and leads to unique structures.

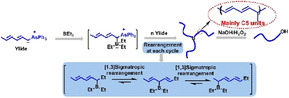

The development of new polymerization methods is an endless temptation for polymer chemists because it provides access to new polymer structures that will offer unprecedented properties and applications. In 1997, Shea et al. discovered a borane‐initiated living C1 polymerization (chains growth by one carbon atom at a time) of dimethylsulfoxonium methylide and coined the name polyhomologation, a totally different method compared to the traditional polymerization of alkenes (C2 polymerization).^[1]^ A lot of studies have proven that the polyhomologation is an effective protocol to synthesize polymethylene‐based homo‐ and block copolymers with different topology.^[2]^ In 2003, Mioskowski et al. developed another borane‐initiated polymerization using 2‐methylallyltriphenyl arsonium ylide as a monomer, which proceeds by successive elongations of three carbon atoms at a time (C3 polymerization).^[3]^ The general mechanism of C3 polymerization is given in Scheme 1. Arsonium ylide and borane initiator react to produce an ate complex, which by rearrangement and simultaneous loss of triphenylarsine forms an allylic borane intermediate. This intermediate undergoes a [1,3]sigmatropic (or boratropic) rearrangement, resulting in an isomeric allylic borane, which after several cycles and oxidation/hydrolysis affords a C3 polymer. Until now, only a few examples of the polymerization of arsonium ylides have been reported.[^3^, ^4^] Therefore, the development of new ylide monomers for the borane‐initiated polymerization is very important.

**Scheme 1:**
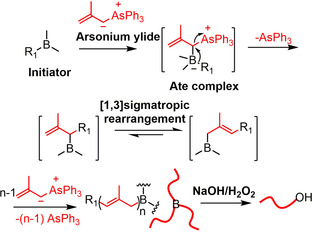
C3 polymerization of 2‐methylallyltriphenyl arsonium ylide initiated by boranes.

In this report, we designed/synthesized three novel dienyltriphenylarsonium ylides and explored their polymerization initiated by triethylborane (BEt_3_) (Scheme 2). Interestingly, it was found that the conjugated double bonds in the dienyltriphenylarsonium ylides cause a significant effect that mainly leads to C5 polymerizations (main‐chain elongations by five carbon atoms of the monomer at a time) and results in new polymeric materials, reported for the first time.

**Scheme 2:**
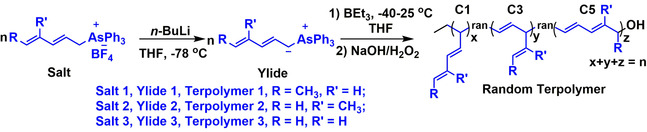
Polymerization of dienyltriphenylarsonium ylides initiated by BEt_3_ followed by oxidation/hydrolysis.

Three dienyltriphenylarsonium ylide salts were synthesized and used as precursors of ylide monomers (see Scheme S1 in the Supporting Information). ^1^H NMR, ^13^C NMR, ^19^F NMR, and ^1^H‐^1^H COSY (Figures S1–S12) confirmed the structures of the obtained dienyltriphenylarsonium ylide salts. Salt 1 exhibited 88.7 % of *E*,*E*‐configuration (*trans*), while salts 2 and 3 had 100 % *E*‐configuration. The deprotonation of the salts by *n*‐butyllithium (*n*‐BuLi) in tetrahydrofuran (THF) at −78 °C under an argon atmosphere, generated in situ the corresponding ylide monomers (ylide 1: dark red solution; ylide 2: light red solution; ylide 3: dark red solution, Figure S13). In order to ensure the complete conversion of salts to ylides, the reactions left overnight.

The polymerization of ylide 1 (with a methyl group at 1‐position, Scheme 2), in the presence of BEt_3_, were carried out using various initial molar ratios of ylide 1 to BEt_3_ ([Yilde 1]_0_/[BEt_3_]_0_=105/1, 150/1, 210/1, and 315/1, Table 1, entries 1–4). The ylide 1 solution was warmed up to 0 °C, BEt_3_ was added, and then the mixture was placed at room temperature immediately. The polymerization occurred with an instantaneous discoloration of the dark red solution indicating the complete consumption of ylide, followed by oxidation/hydrolysis with H_2_O_2_/NaOH, to afford hydroxyl‐terminated polymers (Polymer 1) in 61–78 % yield. The synthesized polymers were characterized by NMR and size exclusion chromatography (SEC). When the initial molar ratios of ylide 1 to BEt_3_ was increased from 105/1 to 315/1, the molecular weight of polymers calculated by both NMR and SEC increased steadily. The SEC traces shift from the low molecular weight to high molecular weight elusion volumes, and they are symmetrical, monomodal, and narrow (*Đ*=1.22–1.27) (Figure 1 b). Besides, the molecular weights of polymers from the NMR are very close to the theoretical ones, as summarized in Table 1. These results indicate the living process of polymerization.

**Figure 1:**
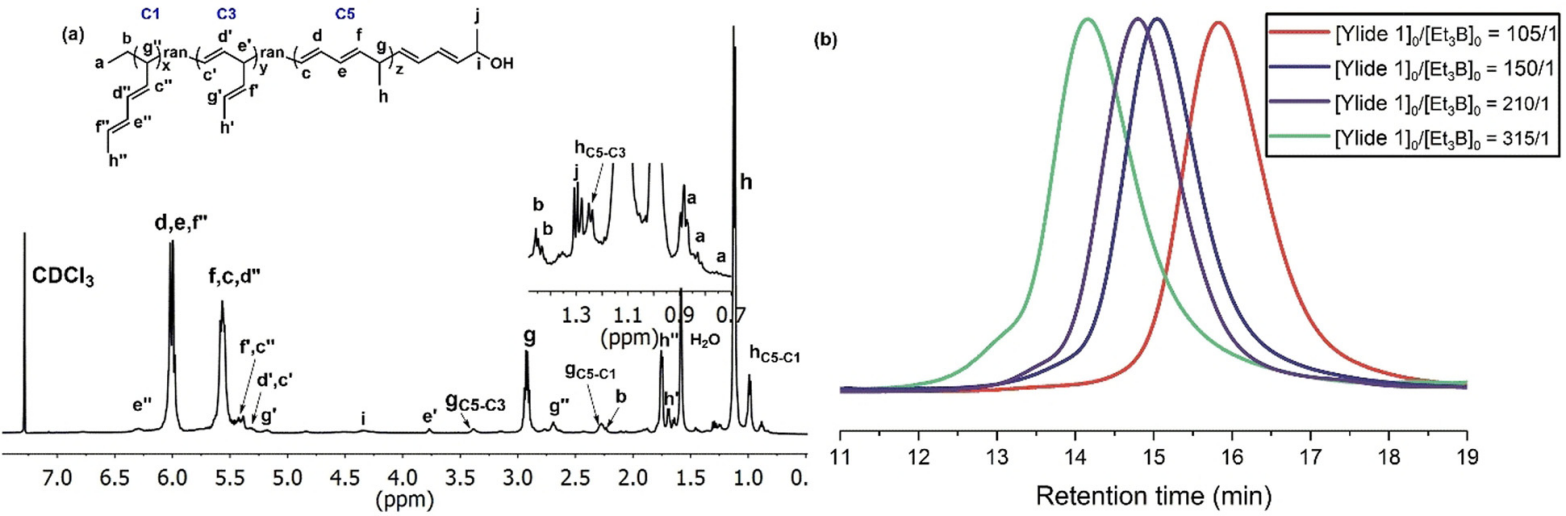
a) ^1^H NMR spectrum (500 MHz, CDCl_3_, 25 °C) of terpolymer 1 (subscript C5‐C3: the connecting part of C5 and C3 segments; subscript C5‐C1: the connecting part of C5 and C1 segments); b) SEC traces of the synthesized polymer 1 with various initial molar ratios of ylide 1 to BEt_3_ ([Yilde]_0_/[BEt_3_]_0_=105/1, 150/1, 210/1, and 315/1, Table 1, entries 1–4) (eluent, THF; flow rate, 1.0 mL min^−1^; 25 °C).

**Table 1:** Polymerization of dienyltriphenylarsonium ylides in the presence of BEt_3_.^[a]^

Entry	Ylide	[Ylide]_0_/[BEt_3_]_0_	*M*_n,theory_^[b]^ [kg mol^−1^]	*M*_n,NMR_^[c]^ [kg mol^−1^]	*M*_n,SEC_^[d]^ [kg mol^−1^]	*Ð* ^[d]^ (M_w_/M_n_)	Yield^[e]^ [%]	C1^[f]^ [%]	C3^[f]^ [%]	C5^[f]^ [%]
1	1	105/1	2.8	2.8	5.5	1.25	61	16.6	3.7	79.7
2	1	150/1	4.1	4.0	9.5	1.25	68	15.5	3.3	81.2
3	1	210/1	5.7	6.8	11.7	1.22	78	14.8	2.7	82.5
4	1	315/1	8.5	14.5	17.3	1.27	77	12.9	3.0	84.1
5^[g]^	2	105/1	2.8	3.1	4.8	1.26	54	17.6	17.1	65.3
6	3	105/1	2.3	3.0	5.1	1.22	66	33.2	4.3	62.5
7	1	51/1	1.4	1.3	3.8	1.18	43	19.3	3.8	76.9

[a] Ylide generation conditions: −78 °C, THF, 12 hours; Polymerization conditions: 25 °C, THF. [b] Determined by the initial molar ratio of ylide to triethylborane. [c] Determined by ^1^H NMR spectroscopy in CDCl_3_, by comparing the integrals of the characteristic signals of methine/methylene adjacent to hydroxy group, at the chain end, to the saturated methine/methylene of all repeating units of the backbone. [d] Determined by SEC in THF using PSt standards. [e] Yield of isolated product. [f] C1, C3, and C5 segment ratio of terpolymers estimated by ^1^H NMR. [g] Polymerization temperature, −40 °C.

A representative ^1^H NMR spectrum of polymer 1 is shown in Figure 1 a. It was observed that the product is a random terpolymer with C1 (one carbon at a time), C3 (three carbons at a time), and C5 (five carbons at a time) repeating units. The proportion of C5 segments located in the main chain estimated by ^1^H NMR varied from 79.7–84.1 % (Table 1). The characteristic signals corresponding to the C5 segments at 5.99–6.04 ppm (d, e), 5.52–5.62 ppm (f, c), 2.89–2.96 ppm (g), and 1.12 ppm (h) were observed. To confirm the main structure of the terpolymer was C5, ^1^H‐^1^H COSY, and FT‐IR measurements were also performed. The peak at 982 cm^−1^ (FT‐IR data, Figure S15) indicates that the double bonds possess the *E*‐configuration (*trans*). By careful analysis of the COSY spectrum (Figure S14), the structure of the terpolymer was further confirmed. It was also observed that the end of polymer 1 is a C5 monomer unit connected to the hydroxyl group, because at the end of the polymerization process, the crowded polymeric organoborane promotes the formation of C5 polymer, which is beneficial for releasing steric hindrance. In addition, continuous C1 or C3 units are never found. This may indicate that the spatial congestion of continuous C1 or C3 units promotes the two [1,3] sigmatropic rearrangements.

The formation of polymer 1 can be explained as described in Scheme 3. An ate complex 2 is initially produced from ylide 1 and triethylborane, followed by 1,2‐migration, with the simultaneous loss of triphenylarsine, to borane 3. The latter may then experience a [1,3] sigmatropic rearrangement that leads to isomeric borane 4, which may undergo a [1,3] sigmatropic rearrangements again to give the isomeric borane 5. During polymerization, one or two [1,3] sigmatropic rearrangements may take place at each cycle, or not, eventually leading to a random three‐armed terpolymer 6, which by oxidation/hydrolysis gives a hydroxyl‐terminated terpolymer 7. It is worth noting that the polymerization would not proceed exactly in the same manner along with the three arms due to the random polymerization. It cannot be excluded that the polymerization may not occur or be slower for one of the three arms,^[4a]^ thus explaining why the molecular weights of polymer 1 are higher than theoretical ones (Table 1, entries 3,4). The higher experimental molecular weights may also be due to the deactivation of a small amount of triethylborane (accidental air oxidation). It was also observed that as the molecular weight of polymer 1 increases from 2.8 to 14.5 kg mol^−1^, the C5 segment ratio increases from 79.7 to 84.1 %, which indicates that high molecular weight induces an important steric crowding, thus facilitating the formation of C5 segments. When the polymerization of ylide 1 was conducted in a lower molar ratio ([Yilde]_0_/[BEt_3_]_0_=51/1, Table 1, entry 7), the C5 segment ratio slightly reduces to 76.9 %, further confirming that high molecular weight and steric crowding promote the formation of C5 segments.

**Scheme 3:**
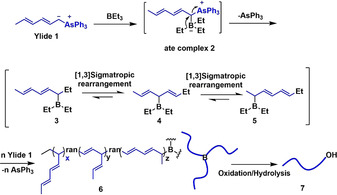
Mechanism of the borane‐initiated polymerization of ylide 1.

The BEt_3_ initiated polymerization of ylide 2 with a methyl substituent at 2‐position and ylide 3 without any substituent (Scheme 2), were also studied (Table 1, entries 5,6). The obtained polymers (Polymer 2, yield, 54 %; Polymer 3, yield, 66 %) were characterized by NMR and SEC. As evidenced by NMR (^1^H NMR and ^1^H‐^1^H COSY, Figures S16–19), polymer 2 and polymer 3 are both random terpolymer with 65.3 % and 62.5 % C5 segments, respectively. The SEC traces are both monomodal and narrow (Polymer 2, *Ð*=1.26; Polymer 3, *Ð*=1.22, Figures S20,21).

Compared to polymer 2 and polymer 3, polymer 1 has the most C5 segments (79.7 %), since the methyl substituent of ylide 1 enhances the electron density of the conjugated double bond, which facilitates the 1,3‐migration on the conjugated double bond. Ylide 2 also has a methyl substituent on the conjugated double bond. Hence, polymer 2 should also have a high C5 segment ratio. However, it is only 65.3 %. This may be attributed to the low polymerization temperature (−40 °C), because ylide 2 is unstable and decomposes above −40 °C. The low polymerization temperature inhibits the [1,3] sigmatropic rearrangement to occur. Even so, the C5 segment ratio of polymer 2 is higher than that of polymer 3 (62.5 %). The C5 segment ratio of polymer 3 is the lowest of the three polymers, which further confirms the electronic effect we proposed above. There is no methyl group (electron‐donating substituent) on the conjugated double bond of ylide 3, which reduces the attractiveness of the 1,3‐migration on the conjugated double bond, resulting in a high C1 segment (33.2 %).

As summarized in Table 1, the C5 segment ratio is the highest, C1 is the second, and C3 is the lowest for all investigated terpolymers. As shown in Figure 2, we obtained the optimized conformations and energy levels of intermediates forming C1 (compound 3), C3 (compound 4), and C5 (compound 5) segments calculated by using the dispersion‐corrected (B3LYP‐D3(BJ)) density functional theory (DFT) method with the 6–311+G(d, p) basis set. The energies of compound 3 are almost the same as that of compound 5, and the energies of compounds 3 and 5 are lower than that of compound 4. This indicates that compounds 3 and 5 have almost the same stability, and they are more stable than compound 4. Since compounds 3 and 5 are the intermediates for producing C1 and C5 segments, the polymerization is prone to form C1 and C5 segments. On the contrary, the C3 segments are less easily formed. Consequently, all terpolymers have the lowest C3 segment ratio (Table 1). In addition, the formation of the C5 segment is beneficial for reducing the crowding of propagating polymeric organoborane, because the C5 segment has no long side chain (only methyl group). Therefore, all terpolymers have the highest C5 segment ratio and the second C1 segment ratio.

**Figure 2:**
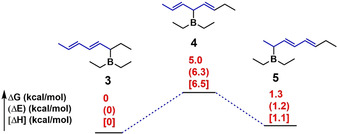
Computed relative Gibbs energy (Δ*G*), electronic energy (Δ*E*), and enthalpy (Δ*H*) by using the dispersion‐corrected (B3LYP‐D3(BJ)) density‐functional theory (DFT) method with the 6–311+G(d, p) basis set.

In summary, the first C5 polymerization is reported, where the main‐chain is growing by five carbon atoms of the monomer at a time. For this purpose, three new dienyltriphenylarsonium ylides were synthesized and polymerized with an organoborane initiator to afford random terpolymers with predominantly C5 repeating units (up to 84.1 %). The outcome of C5 polymerization depends on the occurrence of [1,3] sigmatropic rearrangements, which are related to the substituent of the conjugated double bond of ylides. It has been found that the methyl group (electron‐donating substituent) on the conjugated double bond of ylides facilitates the formation of C5 units. Although the three dienyltriphenylarsonium monomers, synthesized for the first time and used in this study, resulted in random terpolymers, the higher C5 content than C1 and C3 ensures that we are on the right path to pure C5 homopolymerization. Based on these results, we are now designing/synthesizing new ylides and studying their borane‐initiated polymerization.

## Conflict of interest

The authors declare no conflict of interest.

## Supporting information

As a service to our authors and readers, this journal provides supporting information supplied by the authors. Such materials are peer reviewed and may be re‐organized for online delivery, but are not copy‐edited or typeset. Technical support issues arising from supporting information (other than missing files) should be addressed to the authors.

Supplementary
