# Supplementary material for: Boron‐Catalyzed Polymerization of Dienyltriphenylarsonium Ylides: On the Way to Pure C5 Polymerization
Source: Angew Chem Int Ed Engl. 2021 Mar 1;60(15):8431–4. doi: 10.1002/anie.202015217 (PMC8049021; doi:10.1002/anie.202015217)
Supplement: Supplementary file 1 — Supplementary [file ANIE-60-8431-s001.pdf]

Angewandte Chemie
Eine Zeitschrift der Gesellschaft Deutscher Chemiker

Supporting Information

Boron-Catalyzed Polymerization of Dienyltriphenylarsonium Ylides: On the Way to Pure C5 Polymerization

*Xin Wang and Nikos Hadjichristidis**

anie_202015217_sm_miscellaneous_information.pdf

Experimental Details

Materials

All operations of air- and moisture-sensitive chemicals and materials were carried out inflamed Schlenk-type glassware under an argon atmosphere or in an argon-filled glovebox. *n*-Butyllithium (*n*-BuLi) (1.6 M in hexane, Aldrich), triethylborane (BEt_3) (1.0 M in hexane, Aldrich), (2*E*,4*E*)-hexa-2,4-dien-1-ol (97%, Aldrich), triphenylarsine ($AsPh_3$, 97%, Aldrich), phosphorus tribromide (PBr_3, 99%, Aldrich), sodium tetrafluoroborate ($NaBF_4$, 98%, Aldrich), acetonitrile (ACN, anhydrous, Fisher), and diethyl ether (Et_2O, anhydrous, Aldrich) were used as received. (*E*)-penta-2,4-dien-1-ol and (*E*)-4-methylpenta-2,4-dien-1-ol were synthesized according to the literature method.[1] Tetrahydrofuran was distilled over Na and then stored in the glovebox for use. All other chemicals were purchased from Aldrich Chemicals and used as received unless otherwise stated.

Characterizations

Nuclear magnetic resonance (^{1}H NMR, ^{13}C NMR, ^{19}F NMR, and ^{1}H-^{1}H COSY) measurements were recorded on Bruker AVANCE III-400 or 500 MHz instruments. All NMR spectra were taken in $CDCl_3$ unless otherwise stated. Size exclusion chromatography (SEC) analyses were performed using THF as an eluent at a flow rate of 1.0 mL min^{-1} on a VISCOTEK VE2001 system equipped with PSS columns (Styragel HR 2 and 4). The number-average molecular weights ($M_{n,SEC}$) and the molecular weight distribution (M_w/M_n, *Đ*) were obtained by conventional SEC analysis with a calibration curve constructed from polystyrene standards. Fourier transform infrared (FT-IR) spectra were recorded with a NICOLET iS10 FT-IR instrument.

General experimental procedures

Synthesis of dienyltriphenylarsonium ylide salts

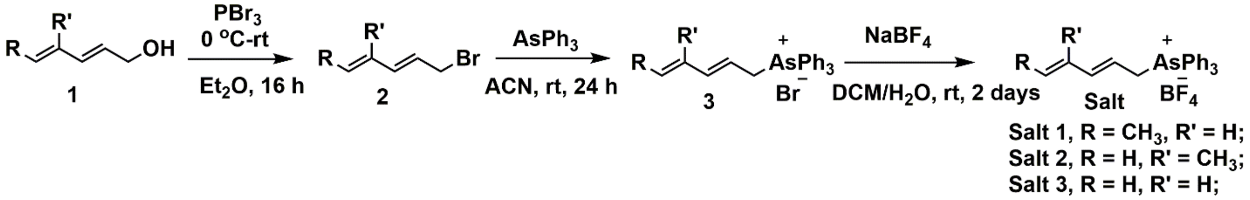

Scheme S1. Synthesis of three dienyltriphenylarsonium ylide salts.

Phosphorus tribromide (4.7 ml, 50 mmol) was added, dropwise at 0 °C under argon, to a solution of (2*E*,4*E*)-hexa-2,4-dien-1-ol (**1**, 9.8 g, 100 mmol) in diethyl ether (200 ml). The reaction mixture was then stirred at room temperature for 16 h, cooled to 0 °C, and quenched with ice water. The organic layer was washed successively with water, saturated sodium bicarbonate, and brine solution. Then, extracted with Et_2O and the combined organic layers were washed with brine and dried over Na_2SO_4, filtered, and concentrated. This crude product **2** was used for the next step without further purification.

2 (10.4 g, 65 mmol) was dissolved in anhydrous ACN (90 ml), followed by the addition of $AsPh_3$ (22 g, 72 mmol). The reaction mixture was then stirred at room temperature for 24 h. The solvent was removed, and the residue was washed with Et_2O to obtain the crude product **3**. **3** (11.2 g, 24 mmol) in 100 ml dichloromethane (DCM) and $NaBF_4$ (79 g) in 90 ml water were mixed and stirred at room temperature for two days. The aqueous layer was extracted with DCM. The combined organic layer was washed with brine and dried over Na_2SO_4, filtered, and concentrated. The crude product was purified by recrystallization (DCM/petroleum ether).

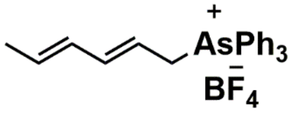

Dienyltriphenylarsonium ylide **salt 1**, white solid, 54% yield, 88.7% *E,E*-configuration, ^1H NMR (500 MHz, $CDCl_3$, ppm): δ 7.58-7.73 (m, 15H, Ar), 6.24-6.29 (m, 1H, d), 5.86-5.91 (m, 1H, c), 5.59-5.66 (m, 1H, b), 5.35-5.41 (m, 1H, e), 4.18 (d, 2H, f), 1.64 (d, 3H, a), see Figure S1; ^{13}C NMR (126 MHz, $CDCl_3$, ppm): δ 140.63, 134.23, 133.22, 132.81, 130.91, 129.82, 120.87, 114.09, 29.67, 18.23, see Figure S2; ^{19}F NMR (471 MHz, $CDCl_3$, ppm): δ -151.88, -151.94, see Figure S3; ^1H-^1H COSY (500-500 MHz, $CDCl_3$, ppm) see Figure S4.

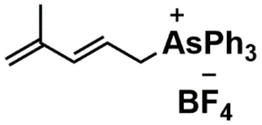

Dienyltriphenylarsonium ylide **salt 2**, white solid, 33% yield, 100% *E*-configuration, ^1H NMR (500 MHz, $CDCl_3$, ppm): δ 7.62-7.75 (m, 15H, Ar), 6.40 (d, 1H, c), 5.47-5.53(m, 1H, d), 4.94 (d, 2H, a), 4.31 (d, 2H, e), 1.66 (s, 3H, b), see Figure S5; ^{13}C NMR (126 MHz, $CDCl_3$, ppm): δ 142.87, 140.39, 134.32, 132.97, 131.01, 121.09, 119.66, 114.42, 29.Fig69, 18.39, see Figure S6; ^{19}F NMR (471 MHz, $CDCl_3$, ppm): δ -151.81, -151.87, see Figure S7; ^1H-^1H COSY (500-500 MHz, $CDCl_3$, ppm), see Figure S8.

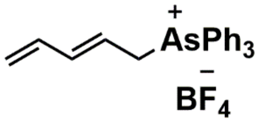

Dienyltriphenylarsonium ylide **salt 3**, white solid, 78% yield, 100% *E*-configuration, ^1H NMR (500 MHz, $CDCl_3$, ppm): δ 7.60-7.75 (m, 15H, f), 6.29-6.35 (q, 1H, c), 6.13-6.22 (m, 1H, b), 5.55-5.62 (m, 1H, d), 5.07-5.16

(2d, 2H, a), 4.26 (d, 2H, e), see Figure S9; ^{13}C NMR (126 MHz, $CDCl_3$, ppm): δ 140.94, 135.10, 134.33, 132.92, 131.01, 120.93, 120.50, 118.01, 29.54, see Figure S10; ^{19}F NMR (471 MHz, $CDCl_3$, ppm): δ -151.74, -151.79, see Figure S11; ^{1}H-^{1}H COSY (500-500 MHz, $CDCl_3$, ppm) see Figure S12.

General polymerization of dienyltriphenylarsonium ylides

A typical C5 polymerization procedure (Ylide 1, Table 1, entry 1) is given below: A suspension of dienyltriphenylarsonium ylide salt 1 (1.52 g, 3.2 mmol) in THF (30 ml) was cooled to -78 ^{o}C. *n*-BuLi (2 ml, 1.6 M in hexane) was then added dropwise to the suspension. Stirring was continued overnight and then for 30 minutes at 0 ^{o}C. To the obtained red solution, triethylborane (30 μL, 1.0 M in hexane) was added quickly, and then the mixture was placed at room temperature immediately. The reaction occurred with an instantaneous discoloration of the solution. After discoloration, H_2O_2/NaOH was added as an oxidizer, and the solution was stirred at room temperature for 4 hours. The organic phase was collected, and the solvent was removed in vacuo. The residue was dissolved in dichloromethane. The polymer was obtained by precipitation in methanol (containing 1 mg / L butylated hydroxytoluene as the antioxidant) three times, where $AsPh_3$ is soluble and therefore easily removed from the polymer. The polymer was dried in a vacuum oven at room temperature for 24 h and then stored in a refrigerator at -40 ^{o}C.

Calculations of C1, C3, and C5 segment ratios

The segment ratios of the three polymers were calculated from the ^{1}H NMR spectra, comparing the integral of the characteristic signal of the corresponding saturated methine/methylene groups to the sum of the integrals of the characteristic signals of C1, C3, and C5 segments.

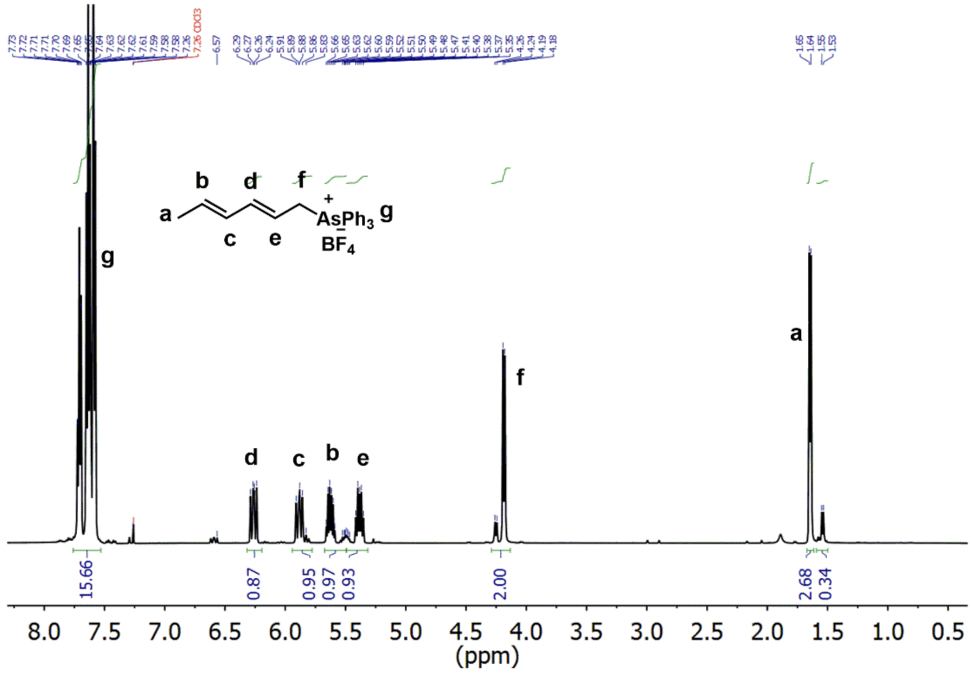

Figure S1. ^{1}H NMR ($CDCl_3$, 25 °C, 500 MHz) spectrum of dienyltriphenylarsonium ylide **salt 1**.

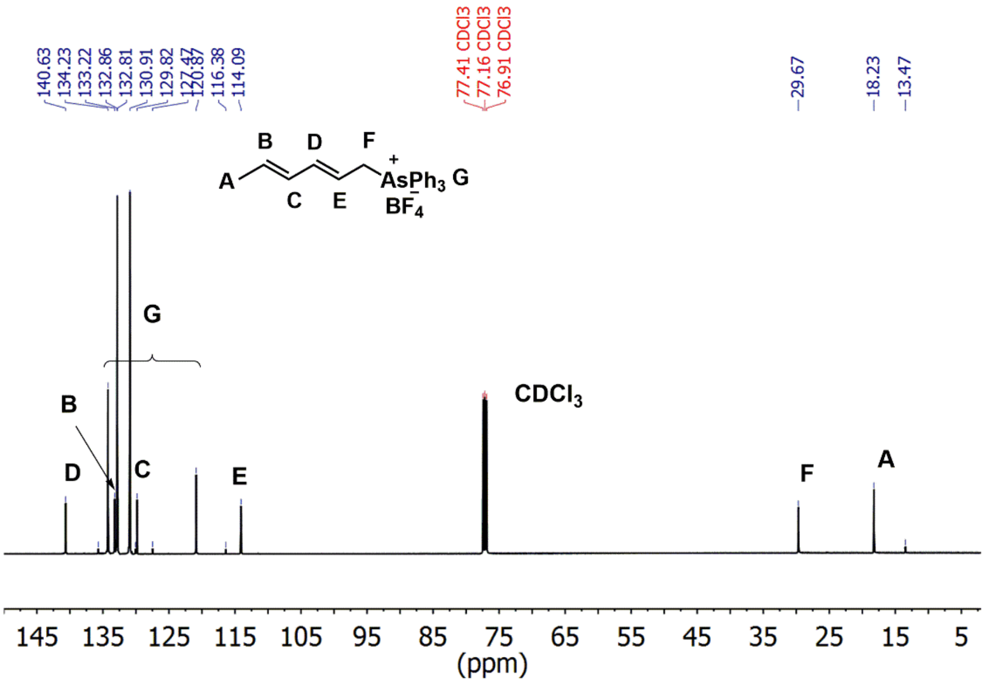

Figure S2. ^{13}C NMR ($CDCl_3$, 25 °C, 125 MHz) spectrum of dienyltriphenylarsonium ylide **salt 1**.

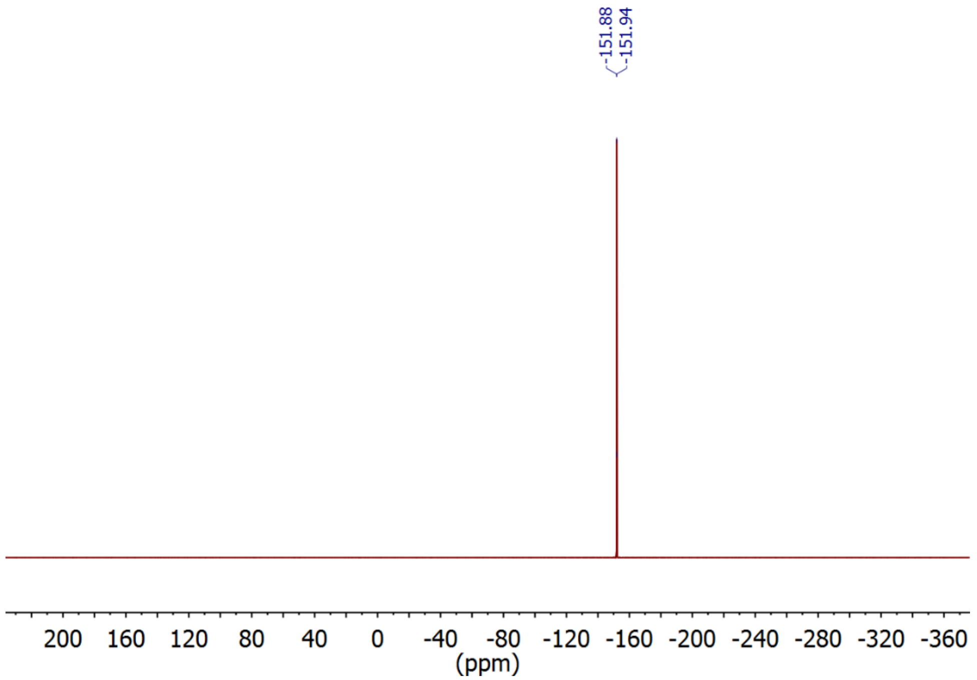

Figure S3. ^{19}F NMR (471 MHz, 25 °C, $CDCl_3$) spectrum of dienyltriphenylarsonium ylide **salt 1**.

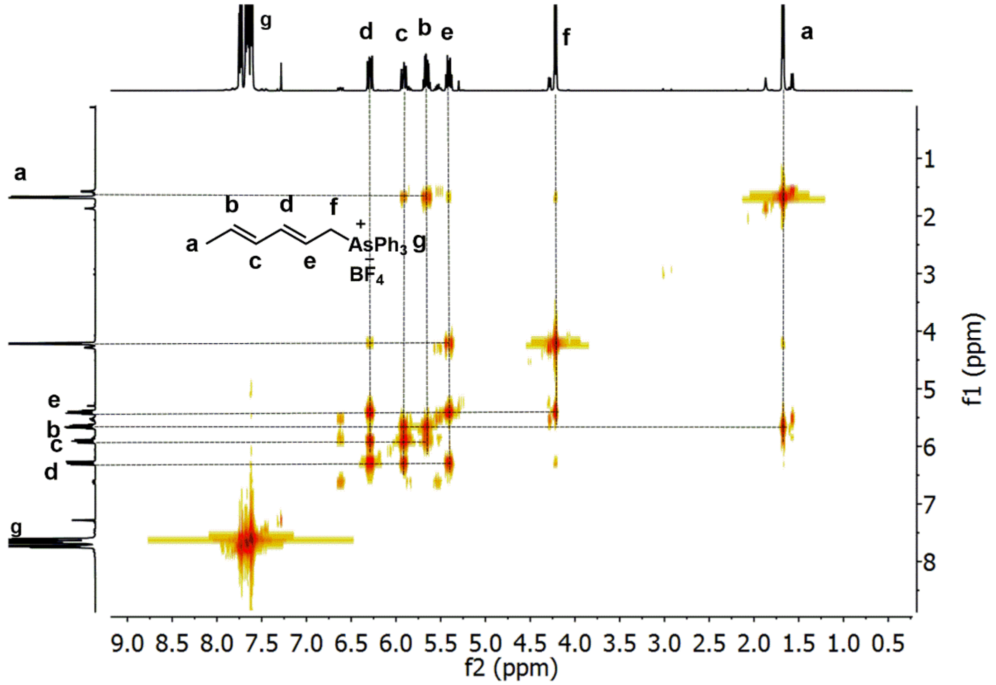

Figure S4. ^{1}H-^{1}H COSY (500-500 MHz, 25 °C, $CDCl_3$) spectrum of dienyltriphenylarsonium ylide **salt 1**.

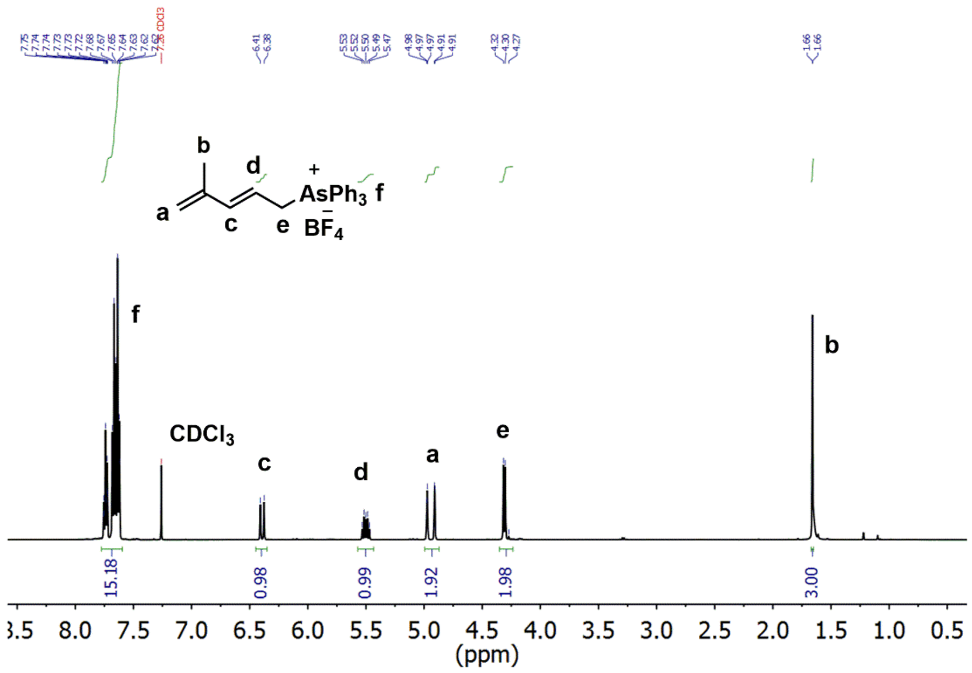

Figure S5. ^{1}H NMR ($CDCl_3$, 25 °C, 500 MHz) spectrum of dienyltriphenylarsonium ylide **salt 2**.

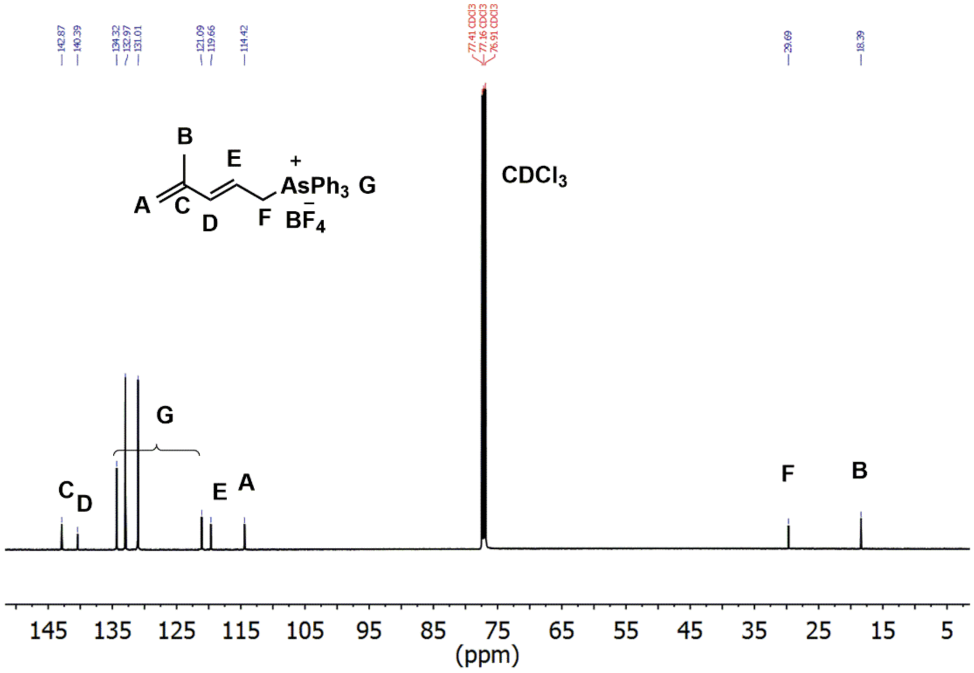

Figure S6. ^{13}C NMR ($CDCl_3$, 25 °C, 125 MHz) spectrum of dienyltriphenylarsonium ylide **salt 2**.

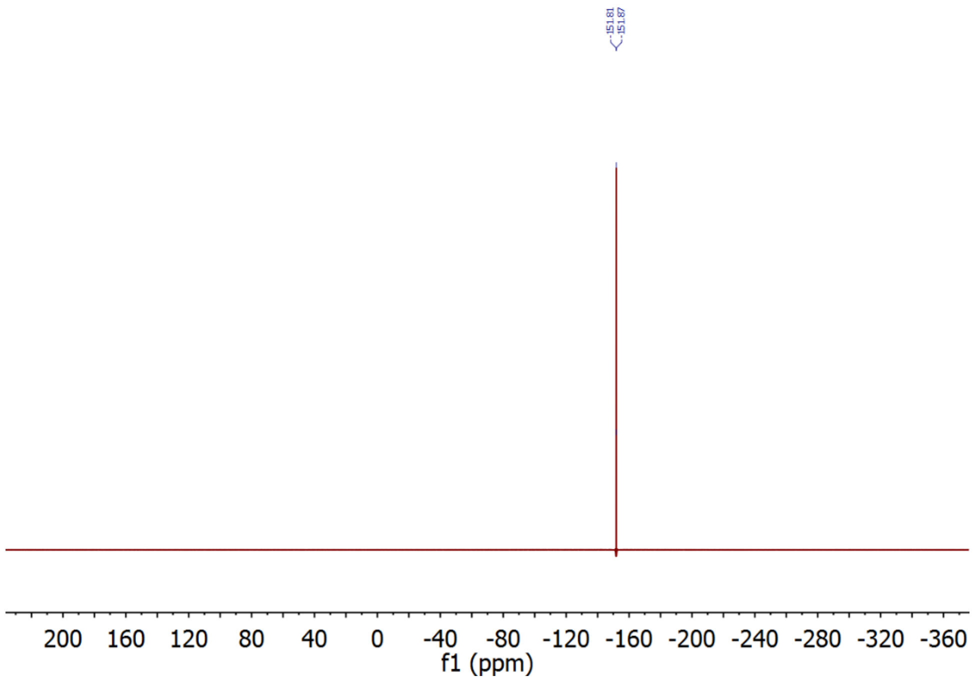

Figure S7. ^{19}F NMR (471 MHz, 25 °C, $CDCl_3$) spectrum of dienyltriphenylarsonium ylide **salt 2**.

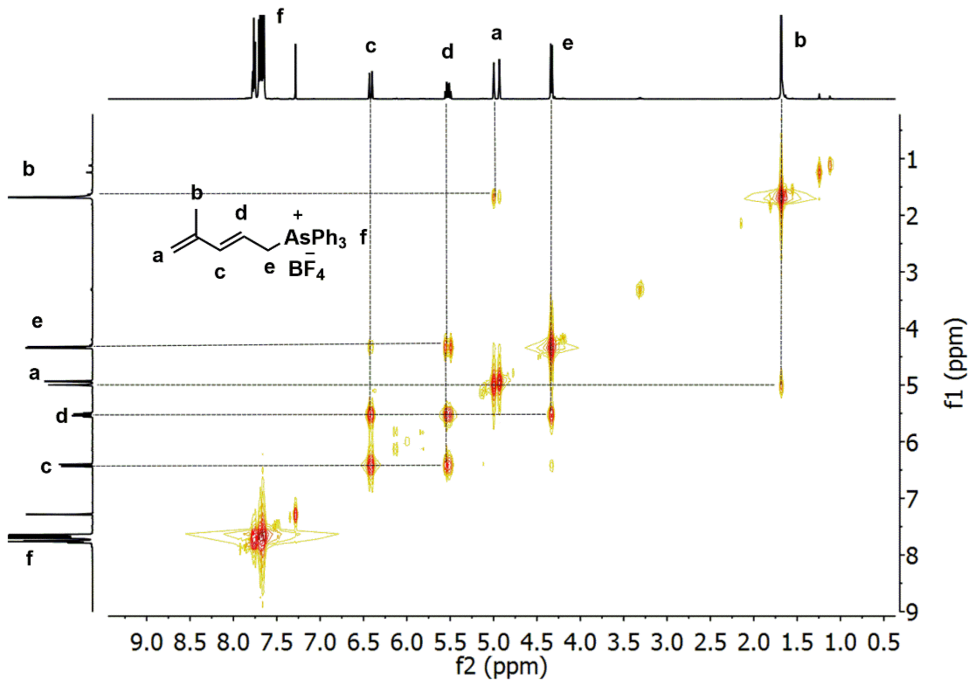

Figure S8. ^{1}H-^{1}H COSY (500-500 MHz, 25 °C, $CDCl_3$) spectrum of dienyltriphenylarsonium ylide **salt 2**.

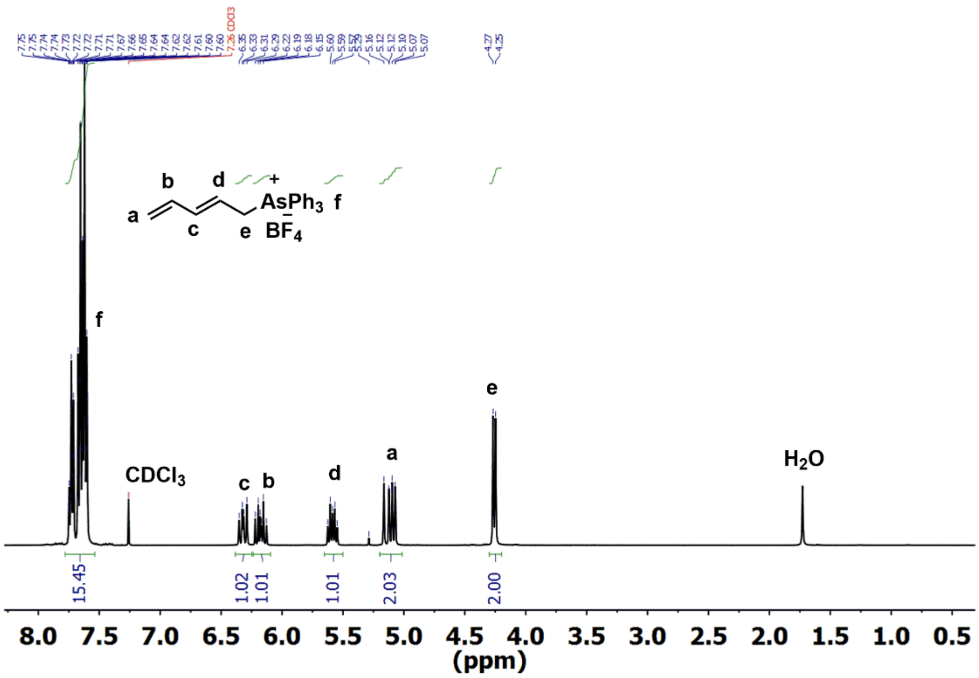

Figure S9. ^{1}H NMR ($CDCl_3$, 25 ^{o}C, 500 MHz) spectrum of dienyltriphenylarsonium ylide **salt 3**.

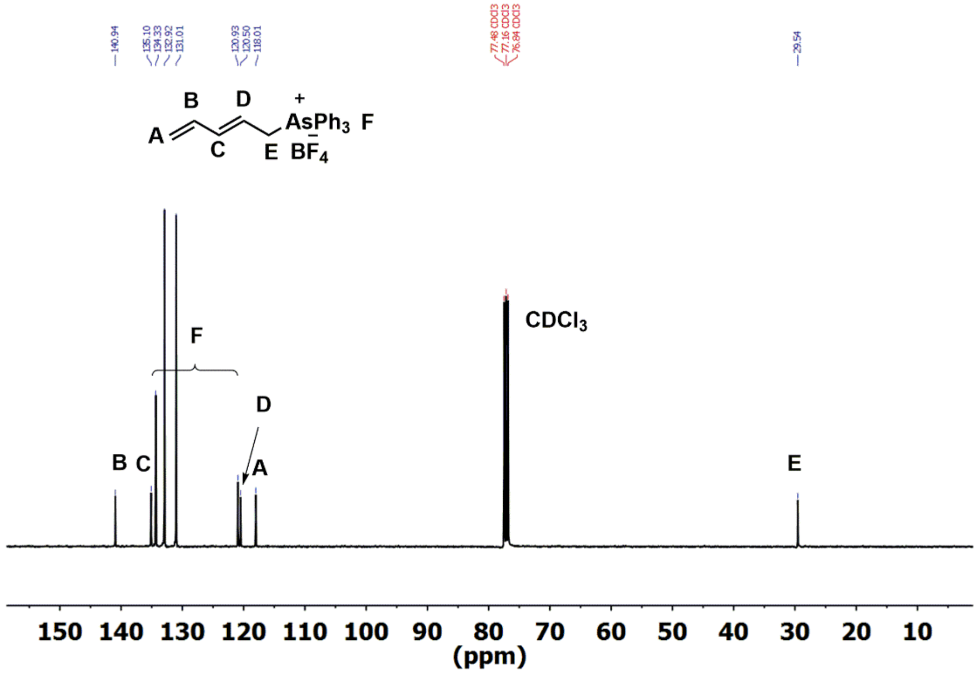

Figure S10. ^{13}C NMR ($CDCl_3$, 25 ^{o}C, 125 MHz) spectrum of dienyltriphenylarsonium ylide **salt 3**.

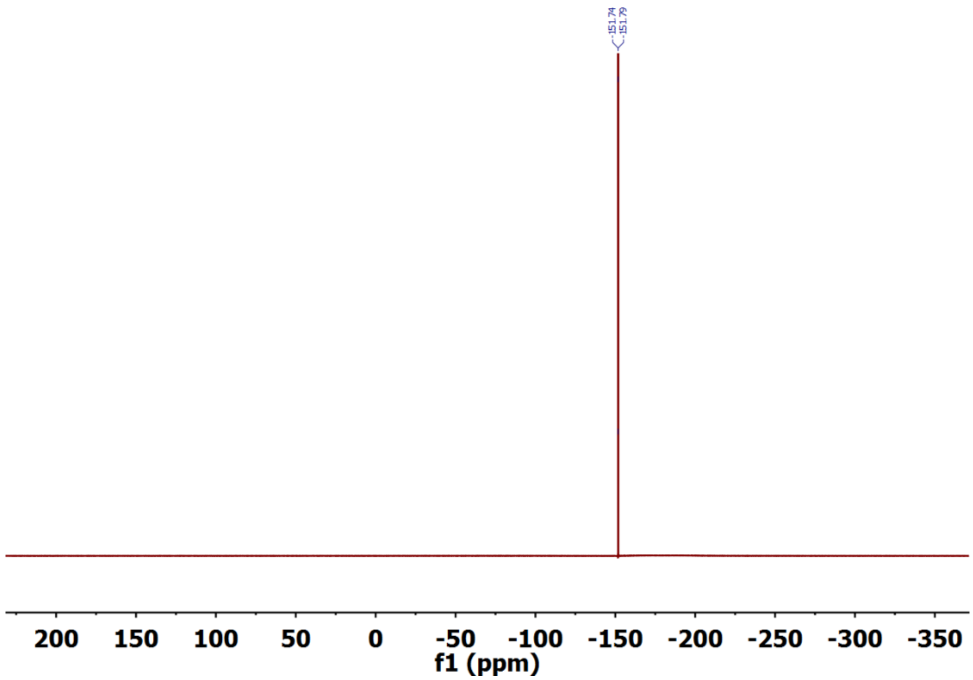

Figure S11. ^{19}F NMR (471 MHz, 25 °C, $CDCl_3$) spectrum of dienyltriphenylarsonium ylide **salt 3**.

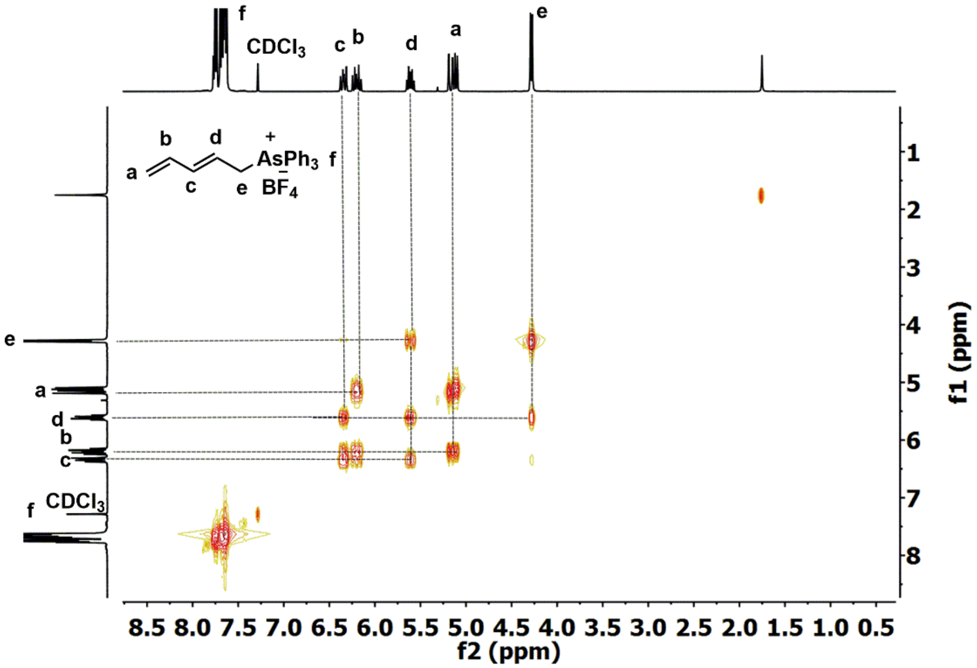

Figure S12. ^{1}H-^{1}H COSY (500-500 MHz, 25 °C, $CDCl_3$) spectrum of dienyltriphenylarsonium ylide **salt 3**.

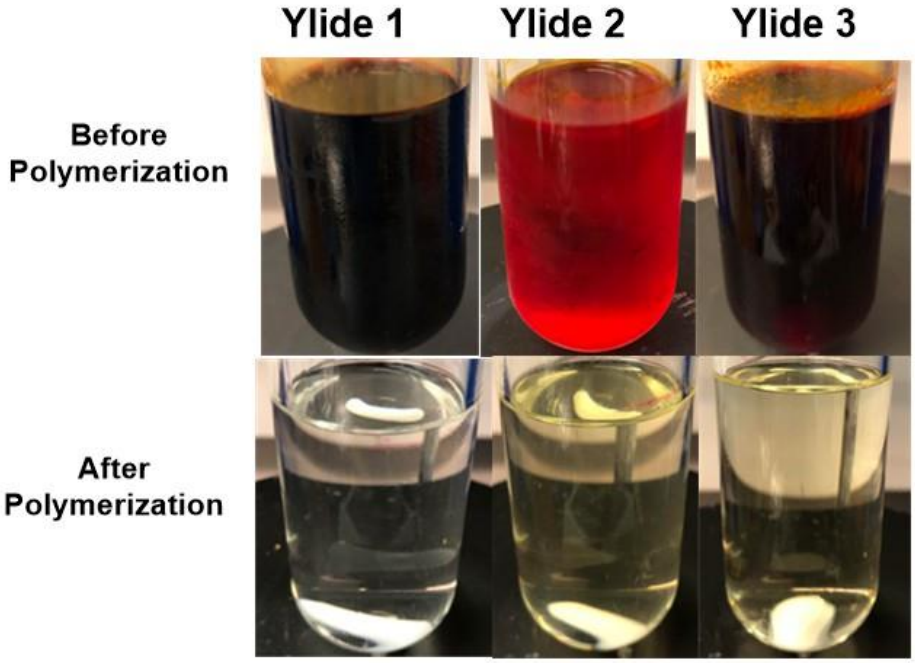

Figure S13. Pictures of the ylide solutions before and after polymerization.

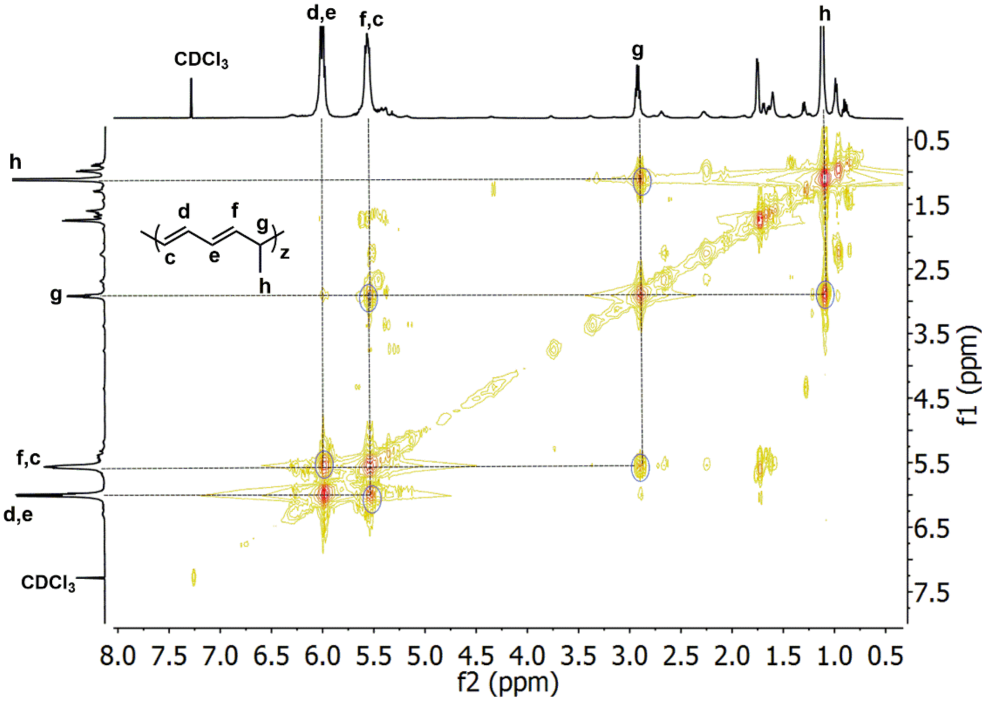

Figure S14. ^{1}H-^{1}H COSY (500-500 MHz, 25 ^{o}C, $CDCl_3$) spectrum of polymer **1**.

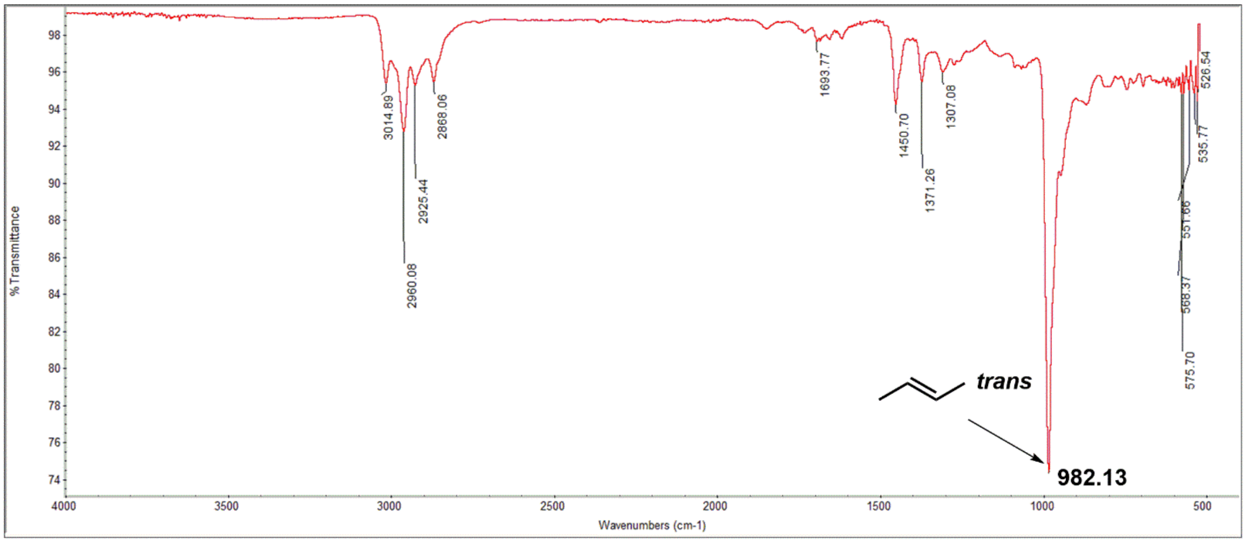

Figure S15. FT-IR spectrum of polymer **1**.

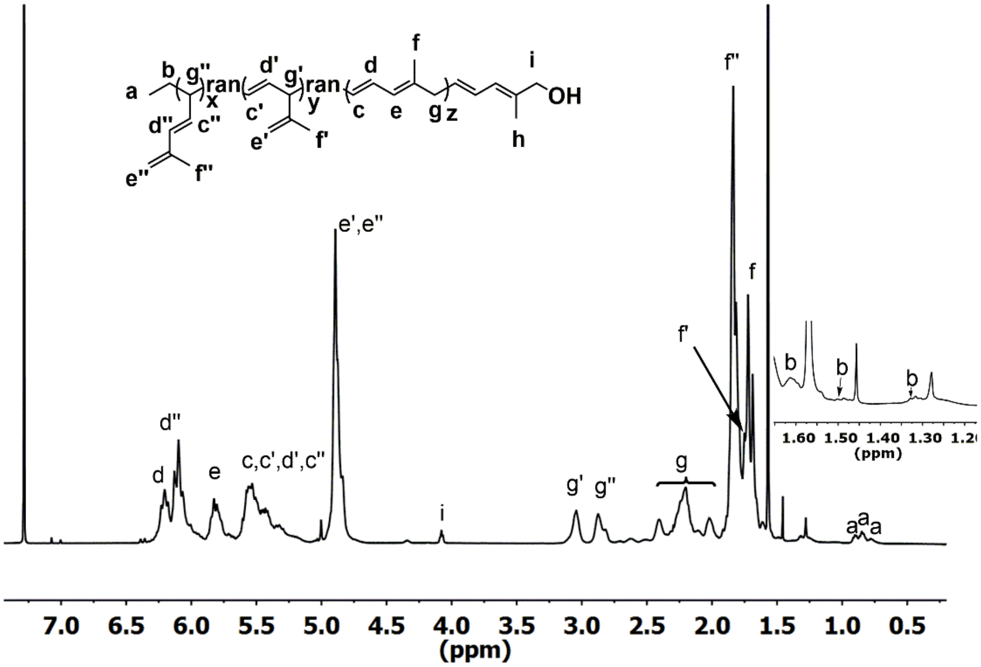

Figure S16. ^{1}H NMR spectrum (500 MHz, $CDCl_3$, 25 °C) of polymer **2**.

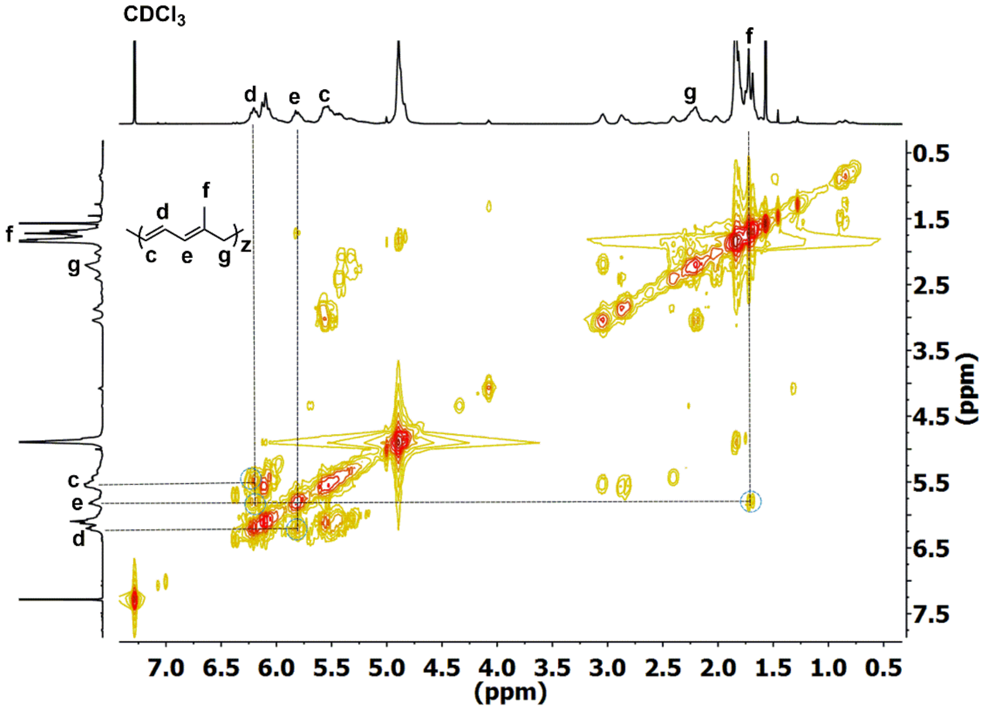

Figure S17. ^{1}H-^{1}H COSY (500-500 MHz, 25 °C, $CDCl_3$) spectrum of polymer **2**.

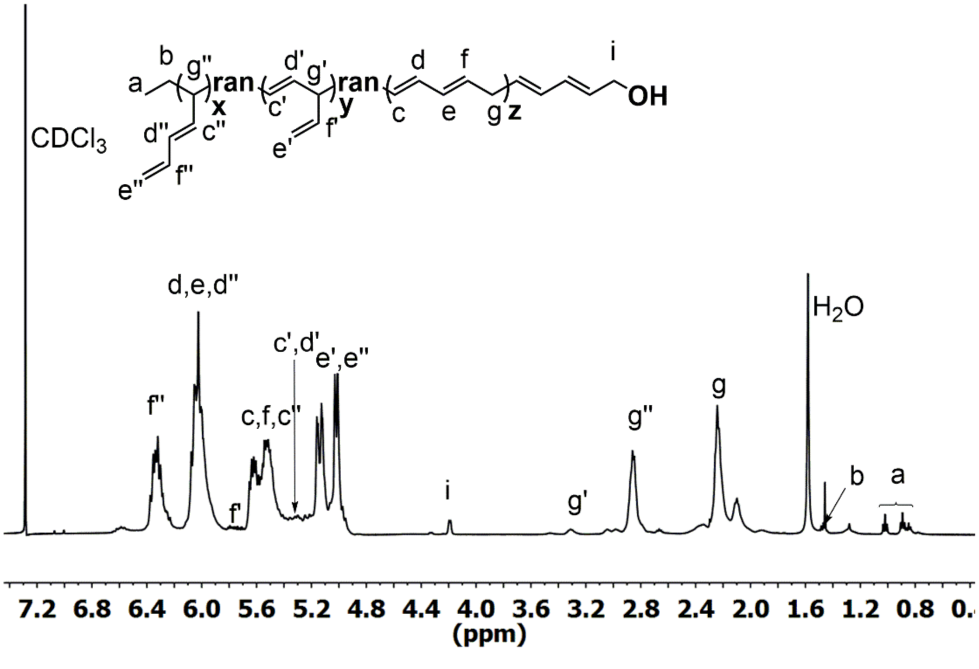

Figure S18. ^{1}H NMR spectrum (500 MHz, $CDCl_3$, 25 °C) of polymer **3**.

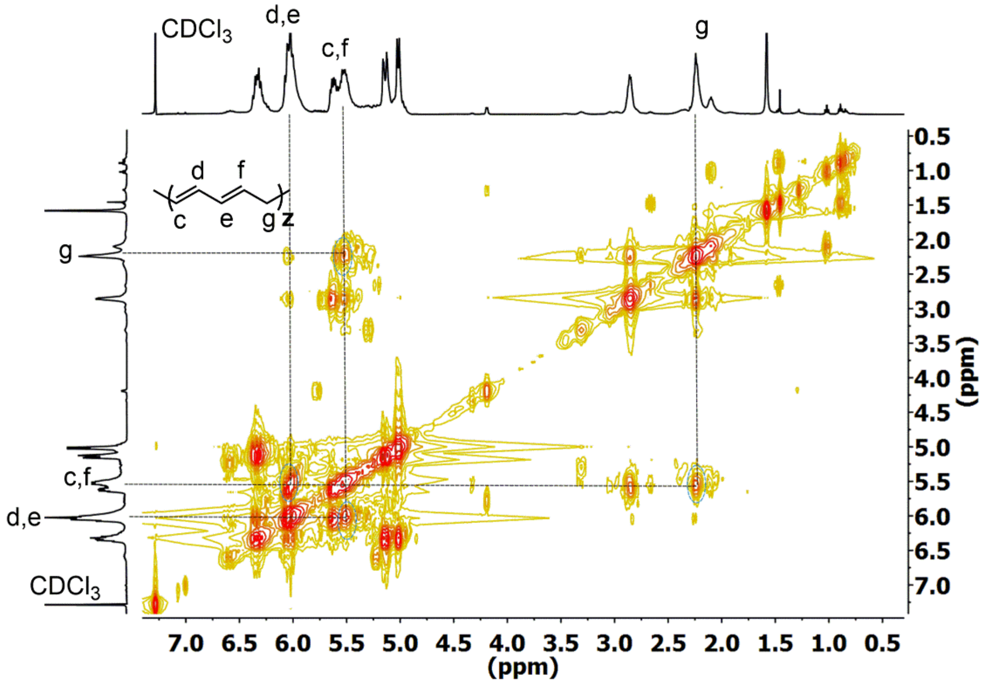

Figure S19. ^{1}H-^{1}H COSY (500-500 MHz, 25 ^{o}C, $CDCl_3$) spectrum of polymer **3**.

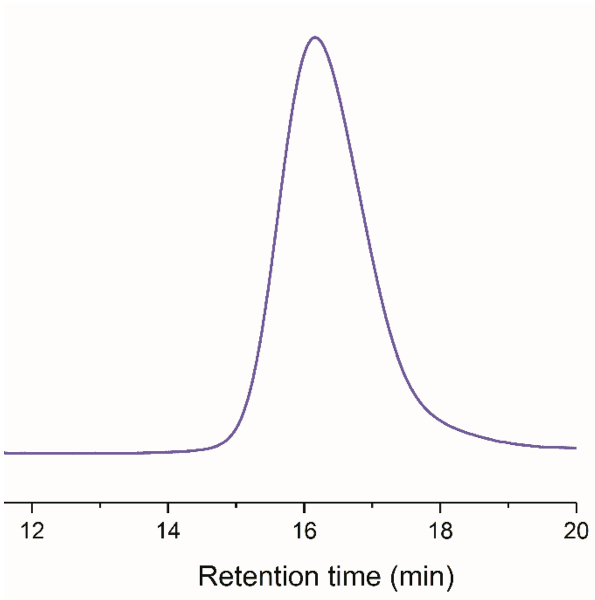

Figure S20. SEC traces of the synthesized polymer **2** ([Yilde 2]$_0$/[BEt_3]$_0$ = 105/1, $M_{n,GPC}$ = 4.8 kg mol^{-1}, $Đ$ = 1.26, Table 1, entry 5) (eluent, THF; flow rate, 1.0 mL min^{-1}; 25 °C).

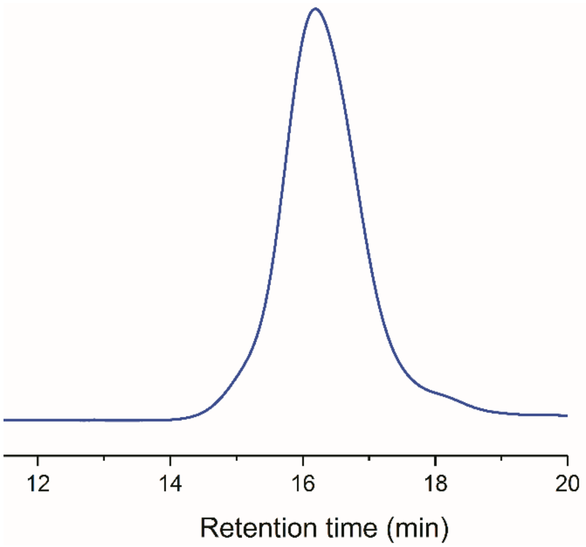

Figure S21. SEC traces of the synthesized polymer **3** ([Yilde 3]$_0$/[BEt$_3$]$_0$ = 105/1, $M_{n,GPC}$ = 5.1 kg mol^{-1}, $Đ$ = 1.22, Table 1, entry 6) (eluent, THF; flow rate, 1.0 mL min^{-1}; 25 °C).

Computational Details

All computations were performed using the Gaussian 09 software package in a Linux multi-processor environment. Geometry optimizations and vibration analysis were performed using the dispersion-corrected (B3LYP-D3(BJ)) density functional theory (DFT) method with the 6-311+G(d, p) basis set. All calculations were performed with the overall molecular charges being zero (Charge = 0) and singlet ground state. Enthalpies and free energies were obtained from the frequency calculations at 298.15 K and 1 atm pressure.To convert Hartree's data to kcal/mol, we used the following conversion factor: 1 Hartree = 627.51 kcal/mol.

Cartesian coordinates (Å) and energies (in Hartree) of the calculated structures

Structure 3 (Figure 2)

Sum of electronic and zero-point Energies= -495.843719

Sum of electronic and thermal Energies= -495.825877

Sum of electronic and thermal Enthalpies= -495.824933

Sum of electronic and thermal Free Energies= -495.891031

1	C	-5.4765660	-0.3758030	0.1139330
2	H	-6.2307820	0.3097230	-0.2892490

3	H	-5.8136350	-1.3895360	-0.1311750
4	H	-5.4711000	-0.2733180	1.2016120
5	C	-4.1267960	-0.0983460	-0.4697880
6	H	-4.0394140	-0.1807840	-1.5521800
7	C	-3.0364600	0.2376220	0.2351660
8	H	-3.1130420	0.3215630	1.3184450
9	C	-1.7340560	0.5003750	-0.3447660
10	H	-1.6554190	0.4115970	-1.4270200
11	C	-0.6430310	0.8469130	0.3615640
12	H	-0.7338420	0.9237930	1.4445310
13	C	0.7383570	1.0585300	-0.2005650
14	H	0.6490990	1.2929520	-1.2680390
15	C	1.5004480	2.1910020	0.5046220
16	H	0.9176120	3.1171590	0.4381990
17	H	1.5924930	1.9651960	1.5727460
18	C	2.8901230	2.4211790	-0.0926810
19	H	2.8220620	2.6709140	-1.1560970
20	H	3.4106180	3.2414060	0.4087660
21	H	3.5151150	1.5275550	-0.0046560
22	B	1.2838670	-0.4233320	-0.0122800
23	C	1.2476770	-1.4004730	-1.2456080
24	H	1.0350850	-2.4350570	-0.9583150
25	H	0.4840730	-1.1047450	-1.9725430
26	C	2.6354300	-1.3518430	-1.9339230
27	H	2.6653130	-2.0004190	-2.8135360
28	H	2.8829460	-0.3381770	-2.2638710
29	H	3.4297730	-1.6785410	-1.2563770
30	C	1.8925560	-0.8633090	1.3750320
31	H	2.9163390	-0.4526370	1.3833060

32	H	1.3951980	-0.3317430	2.1956020
33	C	1.9511210	-2.3662660	1.6821820
34	H	2.5256940	-2.9089780	0.9266510
35	H	2.4150800	-2.5650880	2.6526390
36	H	0.9485730	-2.8036650	1.7009590

Structure 4 (Figure 2)

Sum of electronic and zero-point Energies= -495.833691

Sum of electronic and thermal Energies= -495.815529

Sum of electronic and thermal Enthalpies= -495.814585

Sum of electronic and thermal Free Energies= -495.883064

1	C	3.9113630	-1.7364940	-0.5682980
2	H	4.6781750	-1.3307830	-1.2376340
3	H	4.3897690	-1.8809360	0.4071160
4	H	3.6132050	-2.7163090	-0.9491800
5	C	2.7362040	-0.8113940	-0.4629080
6	H	2.9486810	0.1883920	-0.0859860
7	C	1.4830800	-1.1259410	-0.7891470
8	H	1.2827580	-2.1298320	-1.1659130
9	C	0.3040480	-0.1872920	-0.7394960
10	H	0.1938760	0.2706830	-1.7314300
11	C	-1.0007290	-0.8816730	-0.4157580
12	H	-0.9471820	-1.6848730	0.3158420
13	C	-2.1968170	-0.5330170	-0.8979720
14	H	-2.2548880	0.2816660	-1.6201700
15	C	-3.5018600	-1.1623190	-0.5078950
16	H	-3.9927620	-1.5677830	-1.4016180
17	H	-3.3145140	-2.0098510	0.1590190
18	C	-4.4538470	-0.1646260	0.1704280

19	H	-4.6603050	0.6878610	-0.4834380
20	H	-5.4088160	-0.6364770	0.4170360
21	H	-4.0149400	0.2230680	1.0935120
22	B	0.3017640	0.9606340	0.3658590
23	C	0.0235290	2.4329710	-0.1321250
24	H	-0.2514310	3.1096490	0.6840810
25	H	-0.8117490	2.4355140	-0.8434270
26	C	1.2771040	2.9857290	-0.8502840
27	H	1.5730250	2.3480250	-1.6883750
28	H	1.1015980	3.9904770	-1.2440580
29	H	2.1310950	3.0471270	-0.1686070
30	C	0.6057720	0.7243700	1.8924530
31	H	1.6010290	1.1864960	2.0239410
32	H	-0.0505720	1.3789280	2.4814240
33	C	0.6183620	-0.6828940	2.5035390
34	H	0.9677060	-0.6641470	3.5401530
35	H	-0.3857850	-1.1150760	2.5095360
36	H	1.2659560	-1.3595060	1.9436560

Structure 5 (Figure 2)

Sum of electronic and zero-point Energies= -495.841850

Sum of electronic and thermal Energies= -495.824109

Sum of electronic and thermal Enthalpies= -495.823165

Sum of electronic and thermal Free Energies= -495.889030

1	C	-1.4398710	-0.5011340	-0.9919580
2	H	-1.2560520	0.2014910	-1.8099580
3	C	-2.1266770	-1.7585750	-1.5484540
4	H	-2.3220190	-2.4920020	-0.7612190
5	H	-3.0844380	-1.5103170	-2.0136930

6	H	-1.5021880	-2.2430980	-2.3051630
7	C	-0.1203270	-0.8240300	-0.3370120
8	H	-0.1086210	-1.7078580	0.2998920
9	C	1.0002470	-0.0863370	-0.4250150
10	H	0.9989440	0.8021040	-1.0537730
11	C	2.2405090	-0.3937500	0.2597390
12	H	2.2405940	-1.2798210	0.8932720
13	C	3.3624010	0.3347360	0.1617460
14	H	3.3567670	1.2172910	-0.4779440
15	C	4.6570400	0.0251670	0.8494220
16	H	4.9326320	0.8653810	1.4999680
17	H	4.5278310	-0.8451340	1.5005410
18	C	5.8056490	-0.2285370	-0.1403440
19	H	5.9596050	0.6359060	-0.7928920
20	H	6.7435000	-0.4209190	0.3876870
21	H	5.5865420	-1.0905610	-0.7755400
22	B	-2.1491720	0.2240950	0.2302980
23	C	-2.8579630	-0.6245490	1.3587940
24	H	-2.7386890	-0.1490550	2.3396530
25	H	-2.4470210	-1.6361240	1.4442500
26	C	-4.3721970	-0.7314410	1.0510230
27	H	-4.5553870	-1.2342050	0.0976070
28	H	-4.8941930	-1.2982720	1.8267040
29	H	-4.8387240	0.2564640	0.9967230
30	C	-2.1328860	1.7931830	0.3901900
31	H	-3.1150750	2.1034390	0.7746480
32	H	-1.4542090	1.9967560	1.2335460
33	C	-1.7428080	2.6664230	-0.8086710
34	H	-0.7219790	2.4603760	-1.1403210

35	H	-2.4026480	2.4885210	-1.6633530
36	H	-1.7980130	3.7322270	-0.5688960

References

[1] H. Zhang, S. Q. Ma, Z. Y. Yuan, P. Chen, X. G. Xie, X. L. Wang, X. G. She, *Org. Lett.* **2017**, *19*, 3478-3481.
